# Double burden of malnutrition among households in Ethiopia: a systematic review and meta-analysis

**DOI:** 10.3389/fpubh.2024.1417289

**Published:** 2025-01-30

**Authors:** Mahider Awoke Belay, Eyob Ketema Bogale, Mitiku Tefera Haile, Solomon Ketema Bogale, Eyob Getachew, Getnet Alemu Andarge, Kedir Seid, Gebeyehu Lakew, Amlaku Nigusie Yirsaw, Zenebe Abebe Gebreegziabher, Birhan Ewunu Semagn, Ayenew Takele Alemu

**Affiliations:** ^1^Department of Public Health, College of Medicine and Health Science, Injibara University, Injibara, Ethiopia; ^2^Departments of Health Promotion and Behavioral Science, School of Public Health, College of Medicine and Health Science, Bahir Dar University, Bahir Dar, Ethiopia; ^3^Department of Midwifery School of Nursing and Midwifery, Asrat Woldeyes Health Science Campus, Debre Berhan University, Debre Berhan, Ethiopia; ^4^Department of Nutrition, Antsokiya Gemza Wereda Health Office, Antsokiya, Ethiopia; ^5^Departments of Health Promotion and Communication, School of Public Health, College of Medicine and Health Sciences, University of Gondar, Gondar, Ethiopia; ^6^Bati Primary Hospital, Oromia Special Zone, Bati, Ethiopia; ^7^Department of Epidemiology and Biostatistics, School of Public Health, Asrat Woldeyes Health Science Campus, Debre Berhan University, Debre Berhan, Ethiopia; ^8^Department of Public Health, School of Public Health, Asrat Woldeyes Health Science Campus, Debre Berhan University, Debre Berhan, Ethiopia

**Keywords:** double burden, malnutrition, mother–child pairs, households, systematic review, meta-analysis, Ethiopia

## Abstract

**Introduction:**

The double burden of malnutrition (DBM) at the household level has increased in sub-Saharan African countries as a result of rapid changes in global food systems and growing urbanization. The presence of overweight or obese mothers with undernourished (stunted, wasted, or underweight) children within the same household holds particular significance. However, no comprehensive study or meta-analysis has been conducted to summarize the national evidence of the double burden of malnutrition in mother–child pairs. Therefore, the purpose of this study was to determine the pooled prevalence of the double burden of malnutrition at the household level in Ethiopia in 2024.

**Methods:**

This systematic review and meta-analysis was conducted on the prevalence of the double burden of malnutrition and associated factors among households in Ethiopia, using an advanced search of electronic databases and search engines. The standardized Joanna Briggs Institute (JBI) method was used to extract data from a Microsoft Excel spreadsheet and evaluate the quality of each article. The analysis was performed using STATA V.17. A random-effects model was used to conduct the meta-analysis. Heterogeneity was assessed using the I^2^ and Q tests.

**Results:**

A total of seven publications met the inclusion criteria, including data from 56,877 and 43,770 mother–child pairs for the systematic review and meta-analysis, respectively. The pooled prevalence of the double burden of malnutrition among the mother–child pairs was 8.30% (95% confidence interval (CI): 1.51, 15.09). The heterogeneity test revealed extremely high heterogeneity (I^2^ = 99.91%; *p* = 0.00). In the subgroup analysis based on sample size, the pooled estimated prevalence of the double burden of malnutrition was high for a sample size of fewer than 1,000 mother–child pairs (11.69% (95% CI: 3.11, 20.28)). The pooled estimate from the subgroup analysis of the data collected 8 years ago was 8.61% (95% CI: 1.11, 22.33). Residence, household size, housing quality, wealth index, household food security status, mother’s age and educational status, and child’s age are some of the factors that influence the double burden of malnutrition among mother–child pairs.

**Conclusion:**

In Ethiopia, the double burden of malnutrition among mother–child pairs is rapidly emerging. As a result, double-duty interventions should be implemented to address this issue, taking into account multiple factors at the household level.

**Systematic review registration:**

The protocol for the systematic review and meta-analysis was registered under the registration ID CRD42024517778 with the PROSPERO (International Prospective Register of Systematic Review and Meta-analysis).

## Introduction

The global population is now experiencing the double burden of malnutrition (DBM). The DBM is defined as the simultaneous coexistence of malnutrition due to excess and deficiency in nutrition, including overweight, obesity, undernutrition, and nutrition-related chronic disorders (1). Multiple forms of malnutrition, ranging from undernutrition and micronutrient deficiencies to overnutrition and diet-related non-communicable diseases (NCDs), coexist in communities, families, and individuals due to rapid changes in global food systems and increased urbanization (2–4). The poorest low- and middle-income countries (LMICs) have seen a rise in the DBM, mostly as a result of increasing rates of obesity and overweight (5). One key indicator of the double burden of malnutrition at the household level is the coexistence of an overweight or obese mother and an underweight, stunted, and/or wasted child in the same household (5). Household-level DBM mainly occurs in pairs such as father–child and/or mother–child (6, 7).

Globally, the burden of undernutrition decreased from 900 million (14.7%) people in 2000 to 815 million (11%) in 2016 (3). According to recent estimates, 149.2 million children under the age of 5 were found to be stunted, 45.4 million were found to be wasted, and 38.9 million were found to be overweight (1). The pooled estimates of the DBM among children in 65 low- and middle-income countries (LMICs) were found to be 29.0, 7.5, 15.5, and 5.3% for stunting, wasting, underweight, and overweight/obesity, respectively (8). The pooled estimate of the DBM among mother–child pairs in South and Southeast Asia was reported to be 10.0% (9). In sub-Saharan African (SSA) countries, approximately 8 and 5% of households faced DBM and the triple burden of malnutrition (TBM) among mother–child pairs, respectively (10). The prevalence of the DBM among mother–child pairs was reported to be high in studies conducted in Malaysia (26%) (11), Indonesia (39%) (12), West Java (30.6%) (13), and Argentina (12%) (14). The overall weighted prevalence of the DBM was found to be 1.8% among mother–child pairs in Ethiopia, according to the Ethiopian Demographic and Health Survey (EDHS) report (15).

In addition to being caused by abnormal nutritional absorption, malnutrition (both undernutrition and overnutrition) is also characterized by persistent inflammation and recurrent infections, which may indicate an underlying immunological defect (16). The availability of inexpensive, highly processed foods and beverages, along with the introduction of activity-saving technologies, has led to significant decreases in physical activity at work, at home, and during leisure, primarily contributing to the rise in overweight and obesity (5, 17). The nutrition transition, demographic transition, and epidemiological transition of nations are the three global shifts linked to the causes of the DBM (4).

The DBM has been linked to both undernutrition and overnutrition, with the former leading to increased susceptibility to infectious diseases and impaired childhood development and the latter leading to the increased risk of non-communicable diseases and visceral fat. These health effects have resulted in financial costs for individuals and economies due to decreased productivity, lower wages, and increased medical expenses (18, 19).

Maternal, Infant and Young Child Nutrition (MIYCN) is a complementary strategy to the global action plan on NCDs, which includes targets to reduce obesity and other NCD risk factors, as well as to end all forms of malnutrition by 2030 (20–22). Ethiopia’s National Nutrition Program aims to meet the Millennium Development Goals and address the country’s high rate of malnutrition. National nutrition programs in developing countries should be proactive in addressing both overnutrition and undernutrition, taking into account all aspects of the food and nutrition environment (23). By addressing both issues, this comprehensive approach could prevent the rising issues of OW–OB and associated comorbidities before they become widespread (24). However, one of Ethiopia’s biggest public health concerns and a growing issue is the double burden of malnutrition (25–28). A scoping review was conducted in Ethiopia on the DBM; however, the study included only three publications involving mother–child pairs and did not identify the pooled components (28).

To the best of our knowledge, there are no nationally representative data on the DBM at the household level in Ethiopia. Therefore, this study aimed to determine the pooled prevalence of the DBM at the household level in Ethiopia. The findings contribute to the expanding body of literature on the pooled prevalence of the DBM and offer policymakers additional options for addressing it in Ethiopia to support efforts in achieving the Sustainable Development Goal (SDG), which aims to eliminate all forms of malnutrition by 2030.

## Materials and methods

The entire systematic review was conducted following the Preferred Reporting Items for Systematic Reviews and Meta-analyses (PRISMA) guidelines (29). The techniques and selection of eligible studies were presented using the PRISMA flow diagram (30). The protocol for the systematic review and meta-analysis was registered under the registration ID CRD42024517778 with PROSPERO (International Prospective Register of Systematic Review and Meta-analysis).

### Eligibility criteria

#### Inclusion criteria

Studies conducted in Ethiopia on the double or dual burden of malnutrition and associated factors across all age groups within the household, studies conducted at any time, and studies published in the English language were included. These studies must have reported the prevalence of the DBM [overnutrition (overweight or obesity) or undernutrition (stunting, wasting, underweight, or thinness)] in households or mother–child or father–child pairs. Eligible study designs included cross-sectional (descriptive or analytical), case–control, and cohort studies. Original articles using either secondary data sources or original (primary) surveys, as well as articles that might be published or unpublished, were eligible for inclusion.

#### Exclusion criteria

Studies without full texts, studies published in languages other than English, reviews, studies conducted outside of Ethiopia, and book reviews were excluded.

### Search strategy and data source

To prevent research duplication, PROSPERO registration was initially used to verify whether a prior, comparable study had been carried out. We developed search terms for the PubMed, Cochrane Library, and Health Inter-Network Access to Research Initiative (HINARI) databases. Gray literature was retrieved using the Google and Google Scholar search engines. From March 5 to 7, 2024, a literature search was conducted. The reference lists of the selected studies were examined by contacting experts and using communication addresses that could not be found through search engines and databases.

Article results were limited to human participants and the English language, thereby reducing the number of irrelevant studies found during preliminary searches. The following search keywords were used: “double burden of malnutrition,” “dual forms of malnutrition,” “dual burden of malnutrition,” “determinant*,” “associated factor*,” “mother–child pair*,” “father-child pair*,” “household*,” and “Ethiopia.” Boolean operators (“AND” and “OR”) and truncation (“*”) were applied during the advanced database searching.

### Study selection and data extraction process

Following a detailed search, each study was evaluated by two independent reviewers (MAB and EKB) using the abstracts and titles as a guide. The full text was also evaluated by two other independent reviewers (MTH and SKB). When Cohen’s kappa coefficient was greater than 0.6, the two reviewers’ agreement was accepted. When Cohen’s kappa coefficient was less than 0.6, the screening process was repeated (31). A dual consensus was reached, and a full-text review was conducted. Discussions resolved any disagreements during the review process.

A standardized data extraction format, derived from the Joanna Briggs Institute (JBI) data extraction format generated on a Microsoft Excel spreadsheet, was used by the two independent reviewers (EGD and GAA) to extract data from the selected studies (32, 33). A representative sample of the studies to be examined was used for a pilot test of all data extraction forms, and the results of the test were used to make corrections to the form. Finally, using the structured data extraction format, the two reviewers (KS and GL) independently extracted the following information from the full texts: author, year of publication, study design, region, residence, sample size, participants, prevalence of the DBM, and associated factors.

### Quality appraisal and publication bias in the studies

The quality of the studies was evaluated by the two independent reviewers (ANY and ZAG). The quality of each article, including analytical and descriptive cross-sectional research, was assessed using the standardized Joanna Briggs Institute (JBI) critical evaluation method, which included eight and nine question items, respectively, for each type of study (32, 33). The quality of the results was also evaluated by other two independent reviewers (BES and ATA) after being assessed by ANT and ZAG to check for consistency. The tools consisted of questions with a “Yes” or “No” choice, with a score of one for “Yes” answers and zero for “No” answers. The results were totaled and converted to percentages. For the meta-analysis and systematic review of the prevalence, only studies with a score of 50% or above were considered (32, 33). Low-quality studies were excluded from the review. Any discrepancies in the assessors’ scores were addressed by performing a comprehensive revision, and conflicts were settled through discussion. A funnel plot and Egger’s test were used to assess publication bias (*p* < 0.05 indicated statistically significant publication bias). Publication bias was addressed using the trim-and-fill method for imputation.

### Synthesis of results

The studies found in the selected databases were imported into EndNote, and duplicate files were removed. The retrieved data were transferred to STATA V.17 for further analysis. Heterogeneity was evaluated using the I^2^ test and Q test. An I^2^ value above 75% and a *p*-value less than 0.05 for both the I^2^ test and Q test were considered indicative of significant heterogeneity. According to the interpretation, low, medium, and high heterogeneity were present at I^2^ values of 25, 50, and 75%, respectively (34). The meta-analysis was performed using a random-effects model with the restricted maximum likelihood (REML) method for the test results with heterogeneity. Subgroup and sensitivity analyses were conducted to investigate the potential causes of heterogeneity. A forest plot for proportion with a 95% confidence interval (CI) was used to present the findings of the meta-analysis. Tables and texts were used to display the results of the systematic review and meta-analysis.

## Results

### Study identification

**Figure 1:**
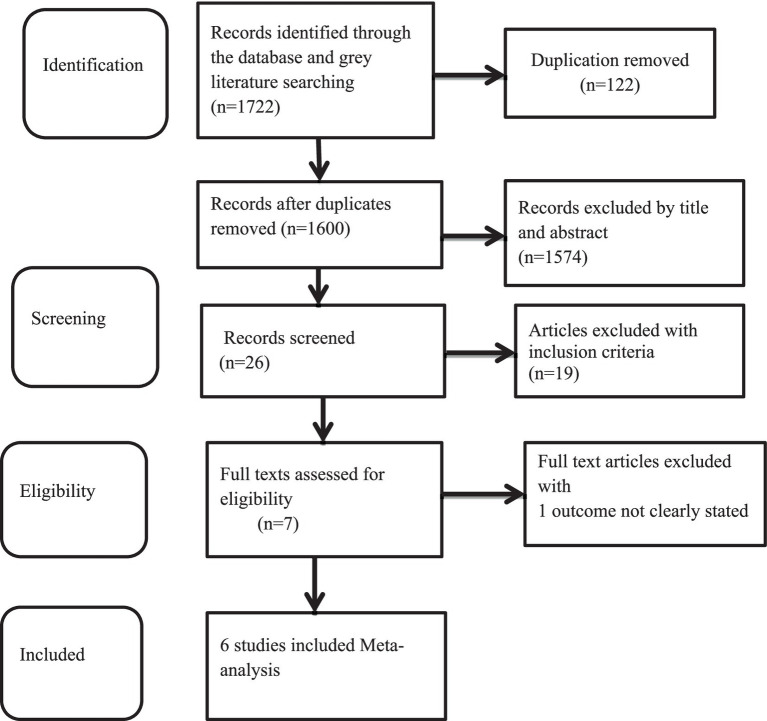
Flow diagram of the studies included in the review of the DBM among mother–child pairs in households in Ethiopia, 2024.

During the database search, a total of 1,722 articles were retrieved from PubMed (776), the Cochrane Library (89), HINARI (35), Google Scholar (518), and Google (304). Of these, 122 duplicate results were removed and 1,574 articles were excluded after screening the titles and abstracts. Finally, we assessed the full texts of the 26 studies for eligibility, and 19 more were excluded based on the inclusion criteria. The remaining seven articles were considered for the systematic review (15, 26, 35–38). One study included two eligible outcome variables and was conducted in Addis Ababa and the rural area of Kersa district in the Oromia region (26). Of the seven studies, six were used to estimate the pooled prevalence of the DBM among households in Ethiopia. One study was excluded from the meta-analysis because it did not report the outcome variable (37) (Figure 1).

### Characteristics of the studies and systematic review

A total of 56,877 and 43,770 mother–child pairs were included in the analysis for the systematic review and meta-analysis, respectively. All studies were cross-sectional. The sample sizes ranged from 577 (35) to 33,454 (38). Three studies used secondary data that were further analyzed by the Ethiopian Demographic and Health Survey (EDHS), conducted in both urban and rural parts of the country (15, 35, 38). The other studies were conducted in Bahir Dar, the capital city of the Amhara region (36); Addis Ababa, the capital city of Ethiopia; and the rural area of Kersa district, Oromia region (26). The highest prevalence of the DBM was reported in the national data of the EDHS 2016, with 22.8% of mothers being overweight or obese with stunted children (35). Similarly, the lowest prevalence (1.52) of the DBM among the mother–child pairs was recorded in the national data of the EDHS 2000–2016 (38) (Table 1).

**Table 1:** Summary of the included studies on the DBM among mother–child pairs in households in Ethiopia, 2024.

S.no	Author	Publication year	Study design	Region	Participants	Sample size	Prevalence of the DBM	Factors affecting the DBM
1	Mekonnen et al.	2024	Cross sectional	Amhara	Mother–child pairs	661	14.5	The child’s DDS, mother’s educational status, wealth index, and household food security status
2	L Bliznashka et al.	2021	Cross sectional	Addis Ababa	Mother–child pairs	592	7.09	Duration of residence, household size, and Orthodox Christianity
3	L Bliznashka et al.	2021	Cross sectional	Oromia	Mother–child pairs	862	2.78	Housing quality, sanitation, and wealth index
4	T. Eshete et al.	2020	Cross sectional	National (EDHS) 2016	Mother–child pairs	577	22.8	Mother’s educational status, residence, and child age
5	Pradeilles et al.	2022	Cross sectional	National (EDHS) 2005–2016	Mother–child pairs	13,107	-	Maternal age, wealth index, number of children, and residence
6	Tarekegn et al.	2022	Cross sectional	National (EDHS) 2016	Mother–child pairs	7,624	1.8	Wealth index, child age, and average birth weight
7	Sahiledengle et al.	2023	Cross sectional	National (EDHS) 2000–2016	Mother–child pairs	33,454	1.52	Short and very short stature

### Prevalence of the DBM among the mother–child pairs in Ethiopia

**Figure 2:**
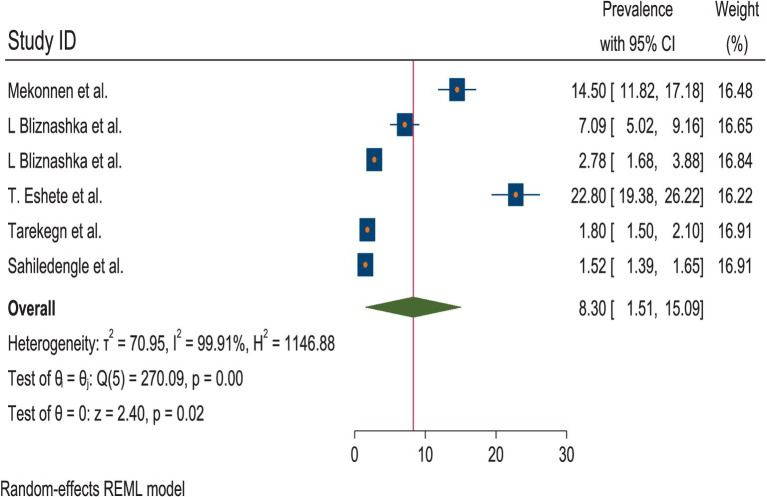
Pooled estimates of the DBM among the mother–child pairs in households in Ethiopia, 2024.

**Figure 3:**
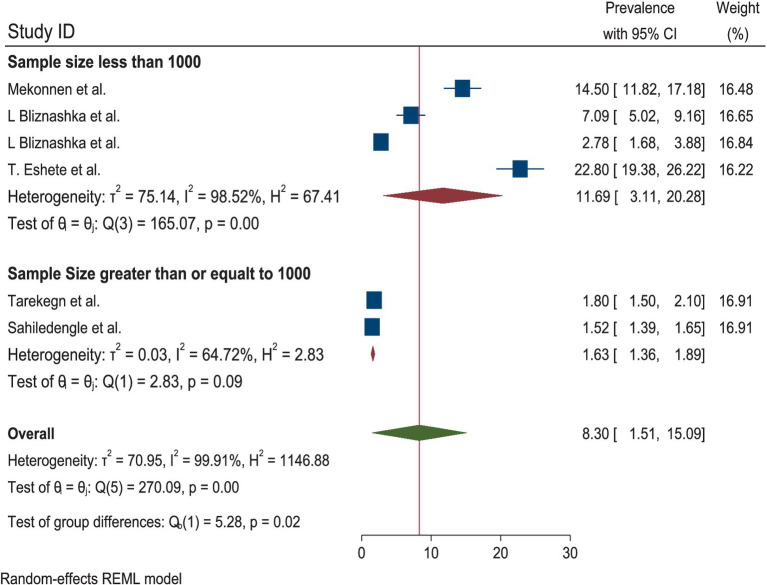
Subgroup analysis by sample size of the DBM among the mother–child pairs in households in Ethiopia, 2024.

**Figure 4:**
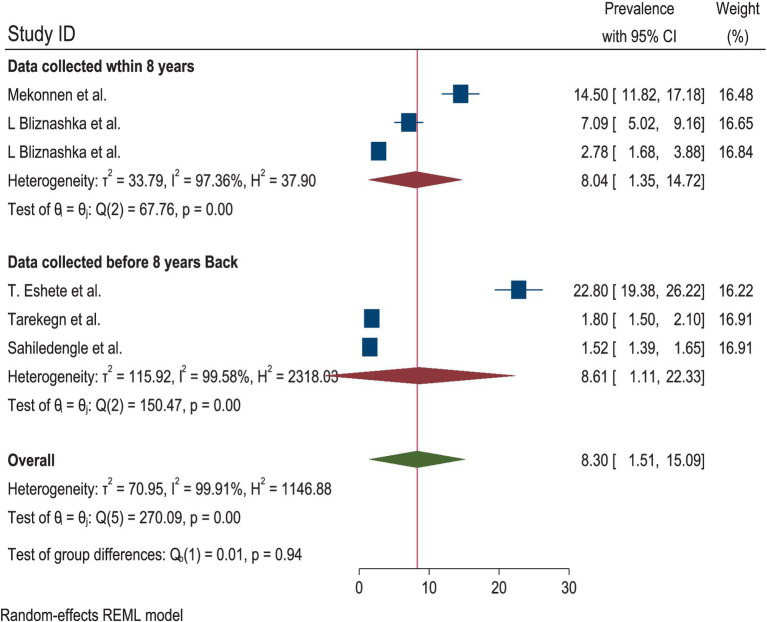
Subgroup analysis by the date of the data collection period for the DBM among the mother–child pairs in households in Ethiopia, 2024.

A total of six studies were included to estimate the pooled prevalence of the DBM among mother–child pairs in households in Ethiopia. Due to the presence of heterogeneity, a random-effects model was employed, and the pooled prevalence of the DBM among the mother–child pairs was 8.30 (95% CI: 1.51, 15.09). Therefore, subgroup analyses were conducted to explain some of the possible causes of heterogeneity. The heterogeneity test for the pooled estimates of the DBM was very high (I^2^ = 99.91% and *p* = 0.00). Subgroup analysis was conducted using sample size and the data collection period. The pooled estimated prevalence of the DBM was high for a sample size of fewer than 1,000 mother–child pairs [11.69% (95% CI: 3.11, 2028)]. The heterogeneity test was also very high (I^2^ = 98.52%, *p* = 0.00). The pooled estimated prevalence of the DBM for the subgroup analysis with a sample size greater than 1,000 mother–child pairs was low [1.63 (95% CI: 1.36, 1.89)]. The heterogeneity test result for this subgroup analysis was categorized as moderate (I^2^ = 64.72%, *p* = 0.09). The pooled estimates of the DBM among the mother–child pairs for the subgroup analysis of the data collection period within 8 years and the data collected 8 years ago were relatively similar: 8.04 (95% CI: 1.35, 14.72) and 8.61 (1.11, 22.33), respectively. The heterogeneity test was very high for both time points (I^2^ = 97.36, *p* = 0.000 and I^2^ = 99.58, *p* = 0.00 for data collected within 8 years and 8 years ago, respectively) (Figures 2–4).

### Quality appraisal, publication bias, and sensitivity tests

**Figure 5:**
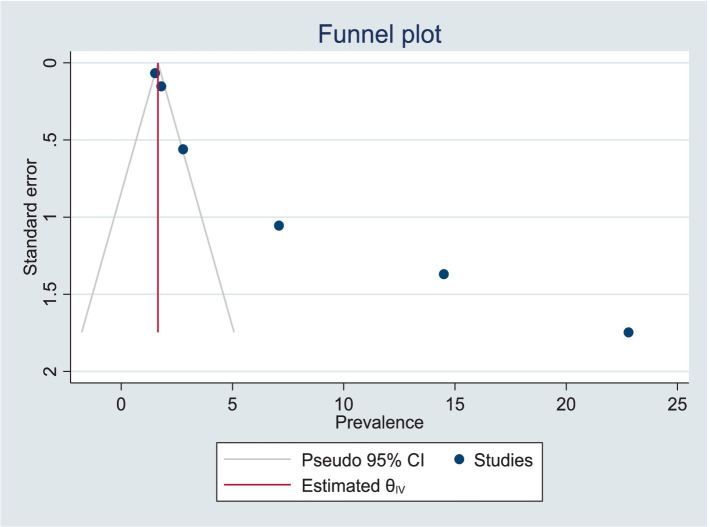
Funnel plot depicting publication bias.

The quality score of all studies was above 80%. The funnel plot displayed an asymmetric distribution, and the Egger’s test yielded a significant result (Prob >|z| = 0.0000), suggesting the presence of publication bias (Figure 5; Table 2).

**Table 2:** Results of the regression-based Egger’s test for small study effects, objectively indicating the presence of publication bias.

Random-effects REML model	
H0: beta	0; no small study effects
Beta1	11.59
SE of beta1	2.075
Z	5.59
Prob >|z|	0.0000

**Figure 6:**
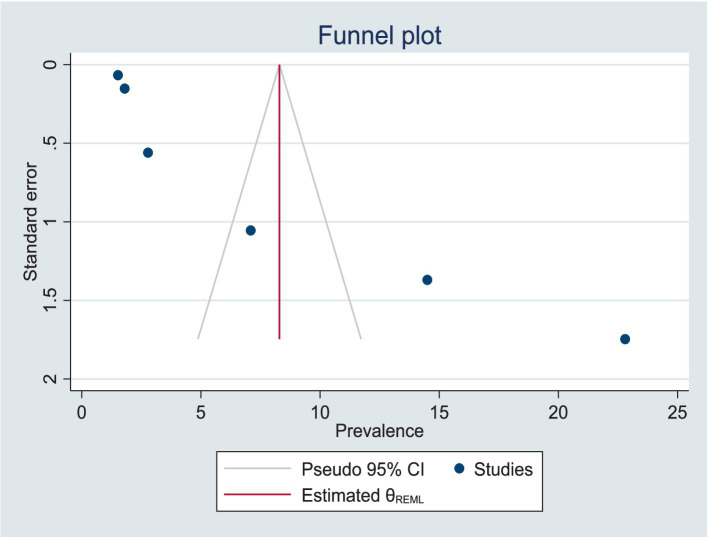
Symmetric presentation of the data after the trim-and-fill method for the analysis of publication bias.

**Figure 7:**
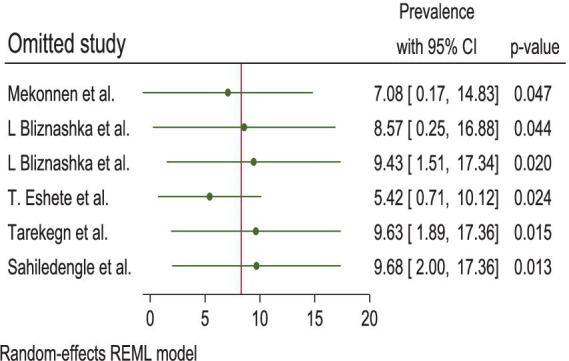
The sensitivity test of the pooled estimates of DBM among mother–child pairs at households in Ethiopia, 2024.

Publication bias was reduced using the trim-and-fill method for analyzing publication bias, with the imputation of zero (0) studies. After imputation, the funnel plot appeared symmetric (Table 3; Figure 6). The sensitivity analysis of the studies showed that no findings affected the overall pooled prevalence, and the *p*-value remained significant (Figure 7).

**Table 3:** Non-parametric trim-and-fill analysis of publication bias for the pooled estimates of the DBM among the mother–child pairs in households in Ethiopia, 2024.

Studies (random-effects REML model)	Prevalence	[95% CI]
Observed = 6	8.296	1.507 15.085
Observed + Imputed (0) = 6	8.296	1.507 15.085

### Factors affecting the DBM among the mother–child pairs in households in Ethiopia

The factors affecting the DBM among mother–child pairs included the duration of residence, household size, Orthodox Christianity, housing quality, sanitation (26), child’s DDS, household food security status (36), mother’s educational status (35, 36), residence (35, 37), maternal age, number of children (37), child age (15, 35), average birth weight (15), wealth index (15, 35–37), and short and very short stature (38). We did not estimate the pooled factors for the DBM among the mother–child pairs in Ethiopia because of the absence of common factors and consistent categories across the retrieved studies.

## Discussion

According to the random-effects model, the pooled prevalence of the DBM among the mother–child pairs in households in Ethiopia was 8.30 (95% CI: 1.51, 15.09). The heterogeneity test for the pooled estimates was very high (I^2^ = 99.91% and *p* = 0.00). Regarding the subgroup analysis, the pooled prevalence of the DBM was higher in the studies with a sample size of fewer than 1,000 mother–child pairs (11.69% (95% CI: 3.11, 2028)). The heterogeneity test was also very high (I^2^ = 98.52%, *p* = 0.00). The pooled estimates from the subgroup analysis of the data collected within 8 years and 8 years ago were relatively similar: 8.04 (95% CI: 1.35, 14.72) and 8.61 (1.11, 22.33), respectively.

In line with the current study, research conducted in various countries has shown the coexistence of overweight or obese mothers with stunted, underweight, or wasted children in households. These studies include those from Ghana (14.2%) (39), sub-Saharan Africa (8%) (10), Bangladesh (6.4%) (40), South and Southeast Asia (12.0%) (9), Colombia (7.8%) (41), Argentina (12%) (14), India (6%) (42), Mexico (8.1%) (43), Philippines (4.3%) (7), Brazil (2.6%) (44), Tanzania (5.6%) (45), and Cameroon (7.7%) (46). Similarly, the DBM prevalence was 4.10, 1.54, 3.93, and 5.54% in Bangladesh, Nepal, Pakistan, and Myanmar (47), respectively. The similarity across these studies may reflect the phenomenon of nutrition transition; the DBM has increased in the poorest low- and middle-income countries. Overweight and obesity primarily increase due to rapid changes in the food system and the availability of cheap processed beverages and foods in LMIC, as well as an increase in leisure-related activities and a reduction in physical activities.

However, the findings of the current study were lower compared to those of studies conducted in Malaysia (26%) (11), Indonesia (39%) (12), West Java (30.6%) (13), Maharashtra (19.7%) (48), Cameroon (16.5%) (46), and Bangladesh (24.5 and 19.8%), where overweight mothers had stunted and underweight children (49). The difference in the result may be due to the current study’s pooled estimates from six different studies, whereas the other studies reported single instances of overweight or obese mothers concurrently with a stunted, wasted, or underweight child. In addition, the difference in the results may be due to variations in the study area, sociodemographic and socioeconomic characteristics, residence, sample size, and study time.

The factors affecting the DBM among the mother–child pairs were identified as the duration of residence, household size, Orthodox Christianity, housing quality, sanitation (26), child’s DDS, household food security status (36), mother’s educational status (35, 36), residence (35, 37), maternal age, number of children (37), child age (15, 35), average birth weight (15), wealth index (15, 35–37), and short and very short stature (38). Since the transition to energy-dense foods, soft drinks, and processed foods leads to increased overnutrition (overweight, obesity, and/or non-communicable diseases), the opposite—dietary inadequacy—aggravates micronutrient deficiencies and undernutrition (stunting, wasting, and underweight).

## Conclusion and recommendations

The findings of the current study reveal that the prevalence of the double burden of malnutrition among mother–child pairs in households in Ethiopia is emerging as a significant public health issue. Therefore, double-duty interventions should be implemented to address the double burden of malnutrition, taking into account various risk factors at the household level. Some of the factors affecting the double burden of malnutrition at the household level included residence, child DDS, wealth index, child and maternal age, educational status of the mother, mother’s stature, average birth weight of the child, household size, number of children, sanitation, and household food security status. Therefore, double-duty interventions, programs, and policies should be implemented to concurrently address the double burden of malnutrition—undernutrition (underweight, wasting, and stunting) and overnutrition (overweight, or obesity)—taking into account these various factors at the household level.

The policy aims to promote, protect, and encourage healthy food choices through legislative measures, communication, and capacity building. As the prevalence of households affected by the double burden of malnutrition continues to rise, the importance of investigating this issue will grow, driving further research in the future. Promoting affordable, well balanced, and healthy meals that align with cultural customs and local food production, along with encouraging the habit of physical exercise, appears to be one of the key strategies for preventing the double burden of malnutrition. The limitations of this study include the small number of studies conducted in Ethiopia, significant heterogeneity, publication bias, and the fact that the available studies for the systematic review and meta-analysis were all cross-sectional. These findings offer policymakers a strategy to support efforts toward achieving the Sustainable Development Goal (SDG) in Ethiopia, which aims to eliminate all forms of malnutrition by 2030.

## Data Availability

The original contributions presented in the study are included in the article/supplementary material, further inquiries can be directed to the corresponding author.
